# Constructing a Ferroptosis-related Long Non-coding RNA Signature to Predict the Prognostic of Head and Neck Squamous Cell Carcinoma Patients by Bioinformatic Analysis

**DOI:** 10.1007/s10528-021-10176-2

**Published:** 2022-01-31

**Authors:** Rui Lu, Zhiyong Li, Shucheng Yin

**Affiliations:** Department of Otorhinolaryngology Head and Neck Surgery, Zhongnan Hospital of Wuhan University, No. 169 Donghu Road, Wuhan, 430071 People’s Republic of China

**Keywords:** Ferroptosis, lncRNA, Head and neck squamous cell carcinoma

## Abstract

Ferroptosis is a novel discovered iron-dependent mode of regulated cell death (RCD) which characterized non-apoptosis. Researches have shown the effect of ferroptosis in the biological activities of tumors. But there is no relevant study showing the relationship between ferroptosis-related genes and head and neck squamous cell carcinoma (HNSCC). In this study, we paid attention to several ferroptosis-related lncRNAs in head and neck squamous cell carcinoma and its clinical features. HNSCC data were obtained from The Cancer Genome Atlas (TCGA) database. Ferroptosis-related lncRNAs were selected by the coexpression network. Then, we identified prognostic ferroptosis-related lncRNAs in HNSCC patients and constructed a ferroptosis-related lncRNAs signature by Lasso cox regression. Besides, GSEA analysis was performed to explore the functional enrichment of ferroptosis-related lncRNAs. Nine ferroptosis-related lncRNAs (AC004687.1, AL450992.2, AC010894.2, AL451085.2, AC104083.1, LIPE-AS1, AC108010.1, CTBP1-DT, and PTCSC2) were identified to have the independent prognostic value in HNSCC patients and the ferroptosis-related lncRNAs signature was constructed based on these nine genes. According to the risk score of the signature, the high-risk group had shorter overall survival (OS) compared with the low-risk group. Risk score showed to be an independent factor for the patients with HNSCC. Additionally, the nomogram was generated and the nine lncRNAs were mainly enriched in phagocytosis, metabolism, and chemokine signaling pathways. The ferroptosis-related lncRNA signature has effects on the prognostic prediction of patients with HNSCC which may serve as potential therapeutic targets for patients with HNSCC.

**Supplementary Information:**

The online version contains supplementary material available at 10.1007/s10528-021-10176-2.

## Introduction

Head and neck squamous cell carcinomas (HNSCCs) encompass various tumors which derive from the mucosal epithelium in oral cavity, pharynx, and larynx in head and neck (Chow 2020). Head and neck cancers rank the 6th most frequent malignant tumor worldwide (Shield et al. 2017) and squamous cell carcinoma is the most common type of head and neck cancer. The incidence of HNSCC increasingly developed over the years and is anticipated to increase by 30% in 2030 (Global Cancer Observatory (GLOBOCAN)) (Ferlay et al. 2019). Epidemiological studies have shown various risk factors of HNSCC, among them the two crucial factors are tobacco and alcohol use, as well as human papillomavirus(HPV) (Rettig and D'Souza 2015). Based on these risk factors, many studies have explored the treatment of HNSCC and progress has been made in targeting therapy (Chow 2020; Koontongkaew 2013; Yang and Deng 2014). But the 5-year overall survival rate remained at about 50%. In recent years, genomics and emerging biomarkers for cancer treatments have invited widespread concerns. Genes including TP53 and CDKN2A and the expression of the E6 and E7 have shown the functional effect in HNSCC. Besides, biomarkers including PD-L1 expression, PD-L2 expression, and other gene signatures showed a potential effect of HNSCC which developed the novel target treatment in patients with HNSCC (Solomon et al. 2018; Leemans et al. 2018; Farmer et al. 2019).

Ferroptosis, which showed the occurrence of iron dependence, is a newly discovered mode of regulated cell death (RCD). RCD is vital in the process of normal development and the maintenance of homeostasis (Dixon et al. 2012; Fuchs and Steller 2011; Degterev et al. 2005). The conception of ferroptosis was first put up by Dixon in 2012 (Dixon et al. 2012). Differentiated from apoptosis or necroptosis at the biochemical level, ferroptosis performed with little relevance to caspase activity and receptor-interacting protein1 (RIPK1) kinase activity (Bebber et al. 2020). Ferroptosis is characterized by the accumulation of lipid reactive oxygen species (ROS) (Dixon et al. 2012; Xie et al. 2016). Besides, it indicated the decreasing of nuclear enzyme GPX4 in the antioxidant system (glutathione system) (Gao et al. 2019; Neitemeier et al. 2017). More importantly, researchers have discovered that cancer cells escaping from other forms of cell death showed sensitivity to ferroptosis (Dolma et al. 2003; Yang and Stockwell 2008; Poursaitidis et al. 2017). Hence an increasing number of current studies paid attention to the mechanism of ferroptosis with cancer (Mou et al. 2019; Liang et al. 2019; Hassannia et al. 2019). Recent studies have suggested that the point of ferroptosis-related SLC7A11(cystine/glutamate antiporter solute carrier family 7 member 11, also known as xCT) served as an HPV-related biomarker of HNSCC with potential therapeutic relevance (Hémon et al. 2020). Obviously, more significant biomarkers of HNSCC are needed to improve the accuracy of prediction and treatment for these patients.

A lot of studies have focused on the genes of the encoding proteins for many years, while long non-coding RNAs (lncRNAs), as a class of non-coding RNA molecules, have received increasing interest these years. LncRNAs have been explored to play important roles in normal cell physiological activities to regulate gene expression levels through complex mechanisms (Ørom et al. 2010; Huarte et al. 2010). Recent evidence has highlighted the association between lncRNA and cancers, including lung cancer (Zhang et al. 2018), hepatocellular carcinoma (Ni et al. 2017), bladder cancer (Cao et al. 2019), as well as head and neck cancer (Luo et al. 2018). LncRNAs were involved in the metastasis of tumors via chromatin remodeling, transcriptional control, and post-transcriptional regulation (Chi et al. 2019). Additionally, current studies have revealed that ferroptosis-related genes play a role in neurodevelopment and the central nervous system (Kim et al. 2021). However, whether ferroptosis-related genes take effect in patients with HNSCC remains unknown. Hence, the analysis combination of ferroptosis-related lncRNA with HNSCC is urgent to be explored, which can lead to a novel biomarker of the HNSCC patients and give a new spotlight on treatment research.

In this report, we concentrated on the effect of several ferroptosis-related lncRNAs with HNSCC and their clinical features. We obtained the data from TCGA and made systematically integrated bioinformatics analyses to identify novel ferroptosis-related lncRNA biomarkers and build a corresponding prognostic prediction model for people with HNSCC. This study further explored the functional enrichment of the biomarkers by Gene Set Enrichment Analysis (GSEA). We demonstrated that these ferroptosis-related lncRNA biomarkers were closely related to HNSCC and have effects on the prognostic prediction of patients with HNSCC.

## Materials and Methods

### Data Collection

The RNA sequencing (RNA-seq) fragments per kilobase of transcript per million (FPKM) data and the corresponding clinical information about HNSCC were downloaded from The Cancer Genome Atlas (TCGA) database (https://portal.gdc.cancer.gov/). The gene expression profiles were normalized using the "limma" R package. Comprehensive gene annotation was performed by the HGNC database (https://www.genenames.org/). Based on recognizing the Ensemble IDs of the genes, lncRNAs were identified according to the gene symbols in the TCGA dataset. HNSCC patients who were diagnosed with head and neck squamous carcinoma and had complete RNA data as well as clinical information were included. Besides, survival data with a follow-up time less than 30 days were excluded (in order to improve the accuracy of our study). We used the programming language (strawberry-perl-5.32.0.1-64bit.msi, https://www.perl.org) to integrate data and extract clinical information. 502 tumor samples were gained from TCGA. There is no need for approval by the ethics committee as TCGA is a public database. The 60 ferroptosis-related genes were obtained from a related study (Hassannia et al. 2019; Stockwell et al. 2017; Bersuker et al. 2019) and attached to the supplementary table *(Supplementary Table S1)*.

### Sorting lncRNA and Ferroptosis-related Genes

The lncRNA was gained from previous normalized gene expression profiles. Ferroptosis-related genes in HNSCC were extracted by "limma" R package. Pearson correlation analysis was implemented to explore the correlation between lncRNAs in HNSCC and ferroptosis-related genes by software R. The square of correlation coefficient∣R2∣ > 0.3 and *p* < 0.001 were significantly considered to be ferroptosis-related lncRNAs. Then, we used Cytoscape software 3.7.2 and software R to visualize the interaction network between lncRNAs and genes.

### Identification of Prognostic Ferroptosis-related lncRNAs

At first, we performed univariate Cox regression to filtrate the prognostic ferroptosis-related lncRNAs in TCGA-HNSC cohort using the “survival” R package. Ferroptosis-related lncRNAs with *p* < 0.05 in univariate Cox regression were selected to the least absolute shrinkage and selection operator (LASSO) regression analysis (with the penalty parameter estimated by tenfold cross-validation) in order to minimize overfitting of the model by the "glmnet" R package. Then, we identified the independent prognostic ferroptosis-related lncRNAs and constructed a multivariate Cox model based on these independent prognostic ferroptosis-related lncRNAs to develop a ferroptosis-related lncRNAs prognostic signature for the HNSCC patients involving ferroptosis-related lncRNAs. The risk score was established to confirm the reliability of identified ferroptosis-related lncRNAs. The risk scores were calculated based on the ferroptosis-related lncRNA expression levels and its corresponding regression coefficient. According to the median risk score, HNSCC patients were divided into a high-risk group and a low-risk group. The risk score was calculated using the following formula:

risk score = $${\sum }_{\mathrm{i}}^{\mathrm{n}}\mathrm{Coefficient}(\mathrm{lncRNAi})*\mathrm{Expression}(\mathrm{lncRNAi})$$.

We used Kaplan–Meier survival analysis and log-rank test to compare the overall survival difference between various groups by the “survival” and “survminer” R packages.

### Constructing Prognostic Model

We established a nomogram by Cox regression using the “rms” R package. The nomogram contained clinical features served to predict the survival of HNSCC patients. The calibration curves indicated the prognostic predictive accuracy of nomogram and index of concordance (C-index) was calculated for the nomogram model. The receiver operating characteristic (ROC) curves and area under the curve (AUC) value were performed to assess the prognostic ability of the nomogram model by “timeROC” R package. The statistical analysis in this study is carried out by R programing language (version 3.6.1, https://www.r-projrct.org).

### Functional Analysis

To further elucidate the biological function enrichment of genes, Gene set enrichment analysis (GSEA4.0.3) was used. The top 5 Gene Ontology (GO) and the Kyoto Encyclopedia of Genes and Genomes (KEGG) pathways related to ferroptosis were visualized by “ggplot2” R package. Gene sets enrichment with nominal *p* < 0.05 and FDR < 0.25 were considered significant.

## Results

### Identify Ferroptosis-related lncRNAs

A total of 14,142 lncRNAs were obtained from the TCGA-HNSC cohort. Among them, the lncRNAs whose expression value was correlated with one or more of 60 ferroptosis-related genes (which mentioned previously) were defined as a ferroptosis-related lncRNA by Pearson correlation analysis (∣R2∣ > 0.3 and *p* < 0.001).

### Ferroptosis-related lncRNA Signature Construction

**Fig. 1:**
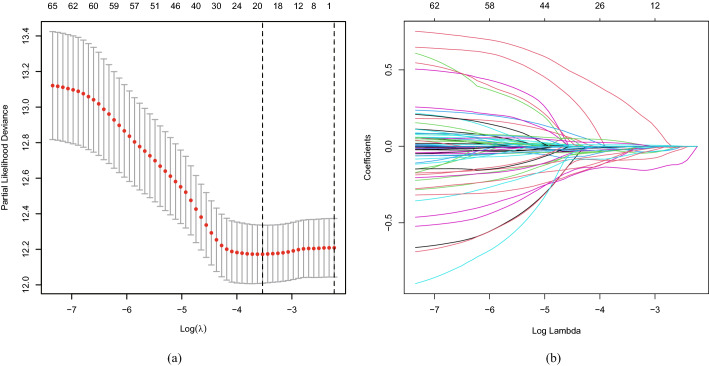
Lasso model based on ferroptosis-related lncRNAs. **a** Lasso coefficient values of 20 ferroptosis-related lncRNAs in head and neck squamous cell carcinoma. The optimal log (λ) value is at the vertical dotted line. **b** Profiles of Lasso coefficients

**Fig. 2:**
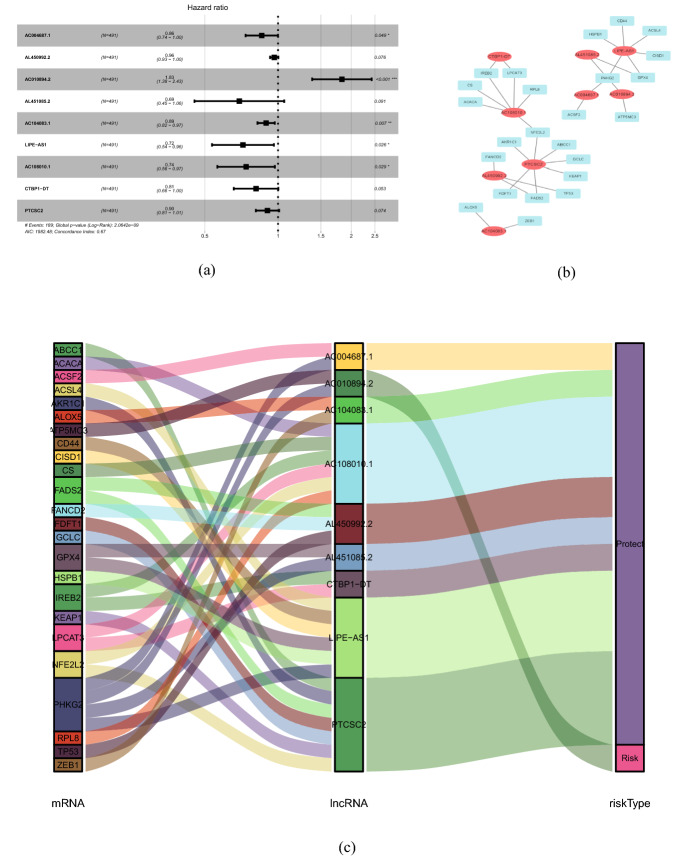
Screening out ferroptosis-related lncRNAs. **a** Ferroptosis-related lncRNAs affect the overall survival of patients. **b** The coexpression network between prognostic ferroptosis-related lncRNAs and ferroptosis-related genes in head and neck squamous cell carcinoma. Red ellipse nodes represent prognostic lncRNAs, and the blue square nodes represent ferroptosis-related genes. **c** Sankey diagram showed the association between prognostic ferroptosis-related lncRNAs, ferroptosis-related genes, and risk types

**Fig. 3:**
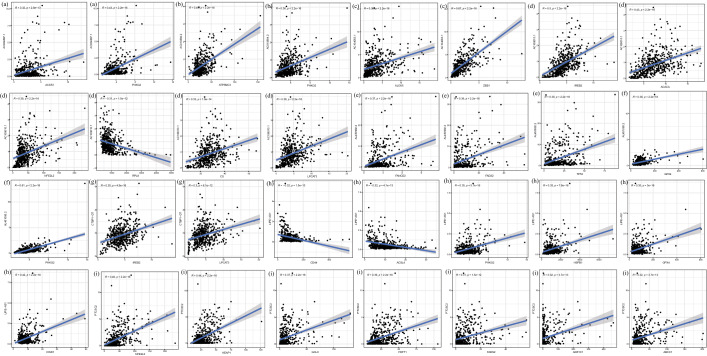
The linear correlations between the nine ferroptosis-related lncRNA and ferroptosis-related genes. **a** AC004687.1 with ferroptosis-related genes. **b** AC010894.2 with ferroptosis-related genes. **c** AC104083.1 with ferroptosis-related genes. **d** AC108010.1 with ferroptosis-related genes. **e** AL450992.2 with ferroptosis-related genes. **f** AL451085.2 with ferroptosis-related genes. **g** CTBP1-DT with ferroptosis-related genes. **h** LIPE-AS1 with ferroptosis-related genes. **i** PTCSC2 with ferroptosis-related genes

**Fig. 4:**
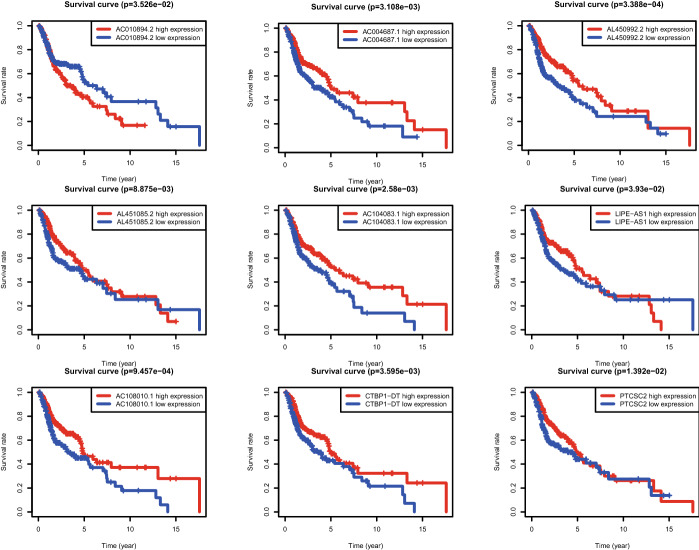
The K-M survival curves of nine prognostic ferroptosis-related lncRNAs. One ferroptosis-related lncRNA (AC010894.2) was a harmful prognostic factor. Eight lncRNAs (AC004687.1, AL450992.2, AL451085.2, AC104083.1, LIPE-AS1, AC108010.1, CTBP1-DT, PTCSC2) were favorable prognostic factors for head and neck squamous cell carcinoma

Combining the clinical information (which contained the information of survival time, survival status, age, gender, grade, stage and TMN), 65 ferroptosis-related lncRNAs were accessed to have a prognostic value of the patients with head and neck squamous cell carcinoma after univariate Cox analysis with *p* < 0.05. Then, we obtained 20 ferroptosis-related lncRNA after Lasso regression (Fig. 1). Furthermore, nine ferroptosis-related lncRNAs showed independent prognostic value in HNSCC according to Multivariate Cox regression (Fig. 2 & Table 1). These nine ferroptosis-related lncRNAs showed varying degrees of linear correlations with ferroptosis-related genes according to Pearson correlation analysis (Fig. 3). Among them, the one lncRNA (AC010894.2) was a harmful prognostic factor, while the others ((AC004687.1, AL450992.2, AL451085.2, AC104083.1, LIPE-AS1, AC108010.1, CTBP1-DT, PTCSC2) were favorable prognostic factors (Fig. 4). We obtained a ferroptosis-related lncRNA signature using the nine lncRNAs. Risk score = – (0.54111 × AC004687.1) – (0.03571 × AL450992.2) + (0.60434 × AC010894.2) – (0.36637 × AL451085.2) –(0.11292 × AC104083.1) – (0.33216 × LIPE-AS1) – (0.30232 × AC108010.1) – (0.20821 × CTBP1-DT) – (0.10038 × PTCSC2).

**Table1:** The nine ferroptosis-related lncRNAs

id	coef	HR	HR.95L	HR.95H	*p* value
AC004687.1	− 0.15411	0.857178	0.735093	0.999539	0.049316
AL450992.2	− 0.03571	0.964919	0.927629	1.003708	0.075751
AC010894.2	0.604346	1.830054	1.380891	2.425318	2.60E-05
AL451085.2	− 0.36637	0.693243	0.453324	1.060137	0.090931
AC104083.1	− 0.11292	0.893219	0.823026	0.969399	0.006846
LIPE-AS1	− 0.33216	0.717371	0.535249	0.961462	0.026217
AC108010.1	− 0.30232	0.739103	0.563012	0.970268	0.029455
CTBP1-DT	− 0.20821	0.812037	0.657662	1.002648	0.052943
PTCSC2	− 0.10038	0.904491	0.810155	1.009811	0.074061

### The Prognostic Effect of the Signature

**Fig. 5:**
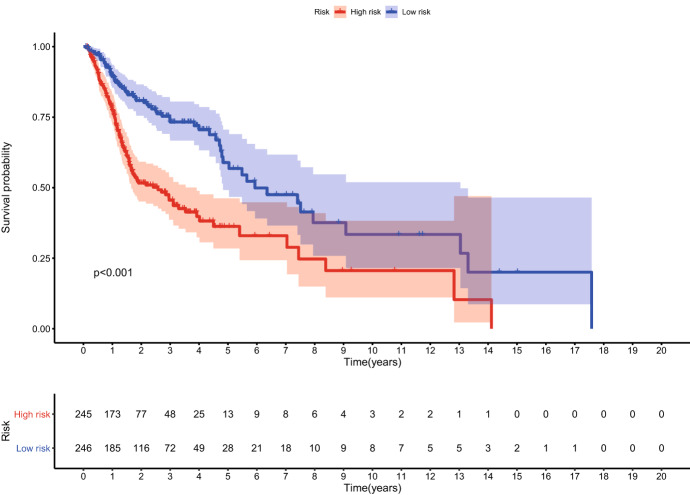
The K-M survival curve of risk score based on nine ferroptosis-related lncRNAs

HNSCC patients in TCGA cohort were divided into a high-risk group and a low-risk group according to the median risk score which was associated with the overall survival (OS) of HNSCC patients. Kaplan–Meier survival curves indicated that HNSCC patients with higher risk score had lower OS rates and a shorter OS time (Fig. 5).

### Clinical Value of the Signature

**Fig. 6:**
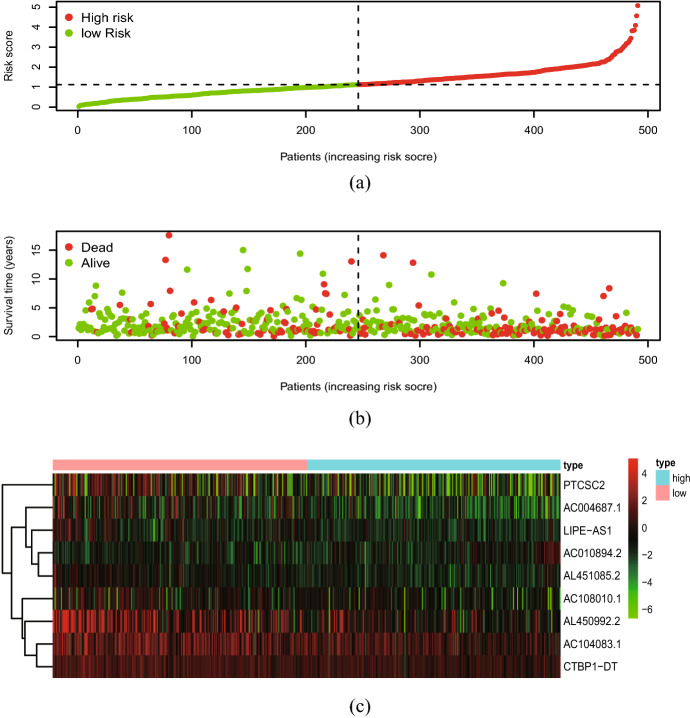
The analysis of ferroptosis-related lncRNAs signature for patients with head and neck squamous cell carcinoma. **a** The risk score between the high-risk group and the low-risk group. **b** The survival time of the patients. **c** Heat map of nine ferroptosis-related lncRNAs’ expression between the high-risk group and low-risk group

**Fig. 7:**
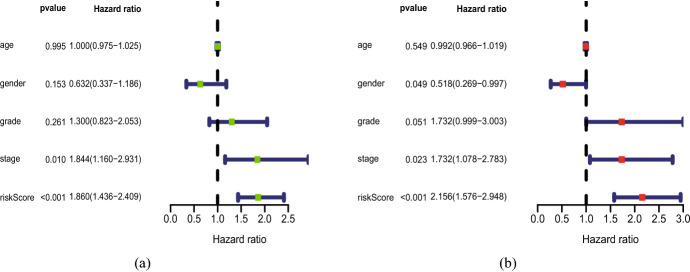
Univariate and multivariate Cox analyses. **a** The forest plots for univariate Cox regression analysis in head and neck squamous cell carcinoma. **b** The forest plots for multivariate Cox regression analysis in head and neck squamous cell carcinoma

Cox regression indicated that risk score had a significant prognostic value. The distribution of the risk score and survival data is illustrated in plot (Fig. 6ab). It is shown that the number of dead patients raised with the increase of risk score. The gene expression profiles of the prognostic risk ferroptosis-related lncRNA between the high-risk group and low-risk group are shown in the heatmap (Fig. 6c). It is indicated that the one lncRNA (AC010894.2) is highly expressed in the high-risk group, while the others ((AC004687.1, AL450992.2, AL451085.2, AC104083.1, LIPE-AS1, AC108010.1, CTBP1-DT, PTCSC2) are highly expressed in the low-risk group. Univariate and multivariate Cox analyses showed that risk score has a significant outcome with *p* < 0.001(Fig. 7). It revealed that risk score was a significant prognostic factor and independent prognostic predictor among patients with HNSCC, while the other factors (age, gender, grade, stage) were not considered to be independent prognostic factors.

**Fig. 8:**
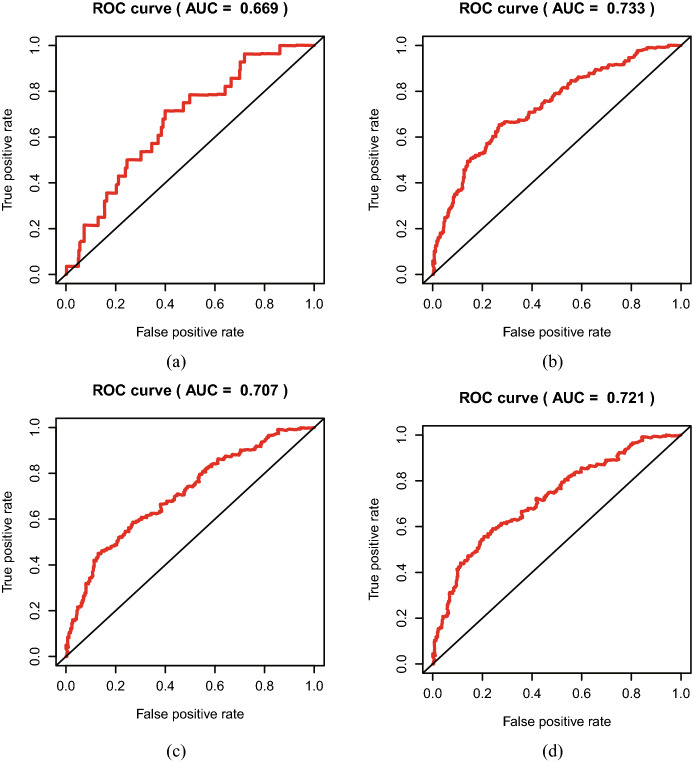
The Receiver Operating Characteristic (ROC) curves of risk score. **a** The areas under the ROC curve about 0.5 year. **b** The areas under the ROC curve about 2 years. **c** The areas under the ROC curve about 3 years. **d** The areas under the ROC curve about 4 years

The Receiver Operating Characteristic (ROC) curves evaluate the predictive performance of the risk score, Area Under Curve (AUC) reached 0.669 at 0.5 year, 0.733 at 2 years, and 0.707 at 3 years, 0.721 at 4 years (Fig. 8), and the maximum AUC value reached 0.733, which indicated good sensitivity and specificity. These demonstrated that the signature harbored a promising ability to predict OS in HNSCC patients.

**Fig. 9:**
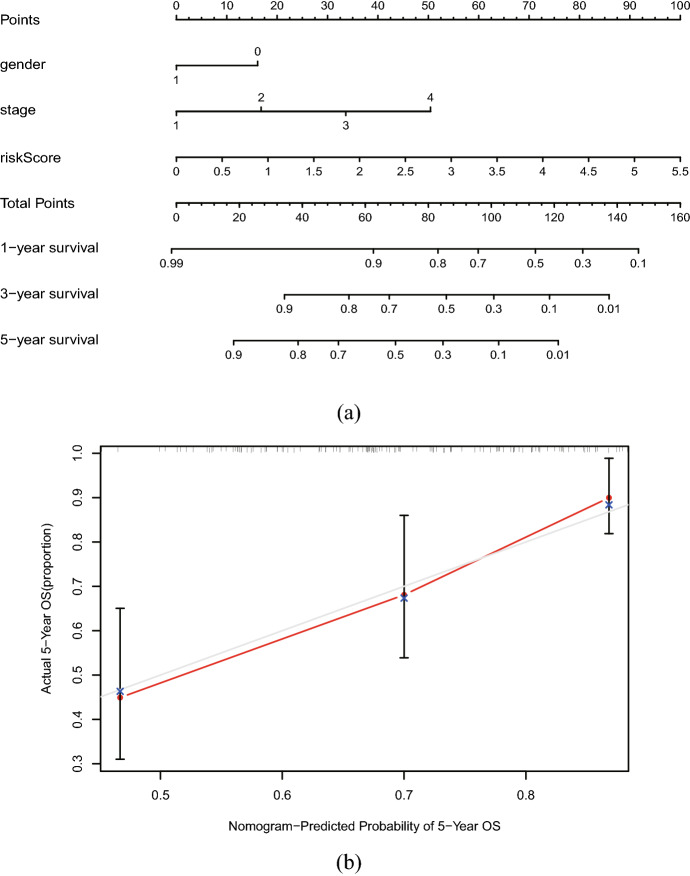
The evaluation of prognostic model based on nine ferroptosis-related lncRNAs. **a** The nomogram of 1-year, 3-year, and 5-year OS based on risk score, gender, and TNM stage. Gender 0 represent female and gender 1 represent male. **b** Calibration plots for evaluating the agreement between the predicted and the actual OS for the prognosis model. The X-axis is nomogram-predicted survival probability and the Y-axis is actual survival probability, respectively. The 45°reference line indicates perfect calibration

**Fig. 10:**
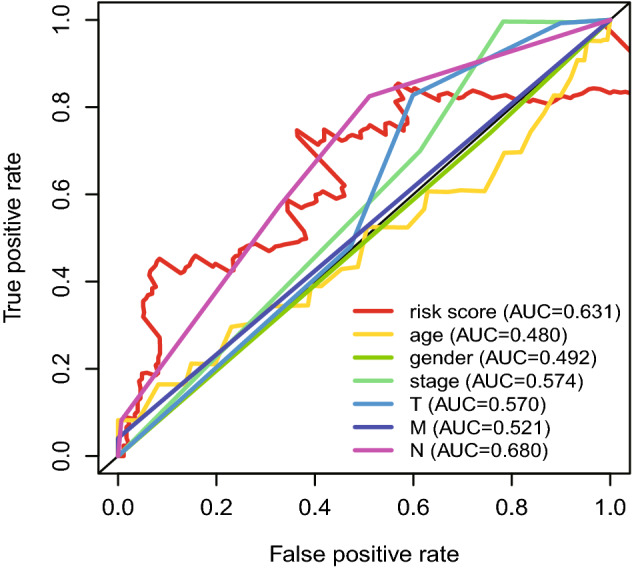
The ROC curves analysis based on risk score and the clinical information

The nomogram was generated which serves as a clinically applicable quantitative tool to predict the OS of HNSCC patients (Fig. 9a), the points of the factors indicate their corresponding contribution to survival probability. C-index of the prognostic model was calculated (0.719) in calibration curves for assessing the predictive ability of the nomogram, and it showed a stable and robust predictive power (Fig. 9b). The actual OS and nomogram-predicted OS matched well at 5 years, as shown by the calibration curves. Compared with the other predictors, the nomogram had excellent accuracy for the five-year survival rate AUC (0.631) of the risk score (Fig. 10). These data reveal that the nomogram has a stable and robust ability to predict the OS for HNSCC patients.

### Functional Analysis

**Fig. 11:**
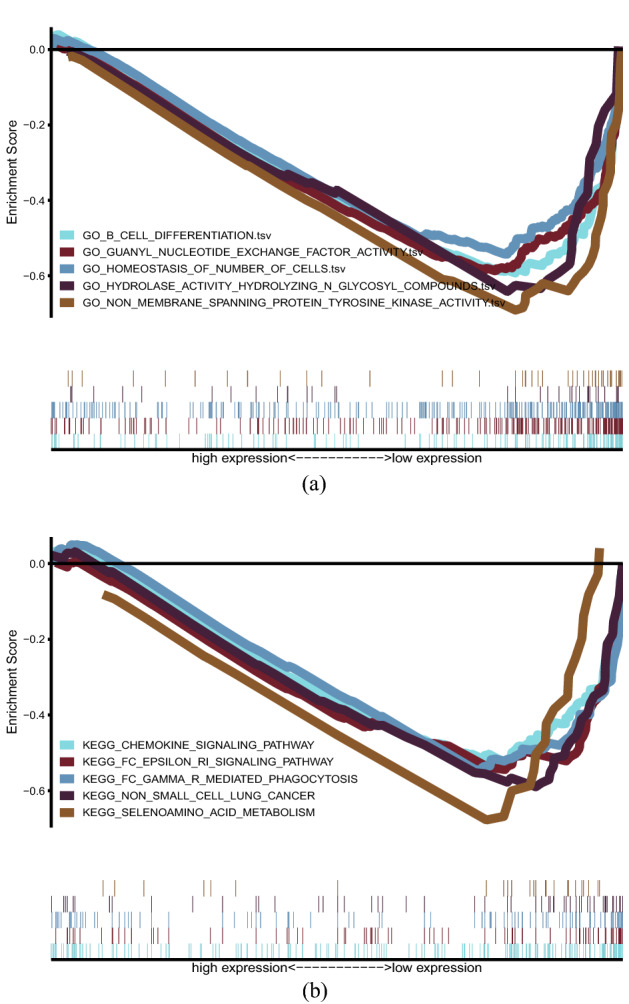
The results of functional analysis. **a** GO enrichment analysis. **b** KEGG enrichment analysis

The ferroptosis-related lncRNAs were mainly involved in hydrolyzing, nucleotide exchange, membrane spanning, cell differentiation, and homeostasis process by GO analysis (Fig. 11a), while KEGG pathways indicate that the lncRNAs were mostly concentrated in phagocytosis, metabolism, and chemokine signaling pathways (Fig. 11b). These biological pathways have a significant effect on the tumorigenesis of HNSCC.

## Discussion

Ferroptosis as a programmed cell death has crucial effects on the occurrence and progression of tumors. Ferroptosis-related genes signature has been explored to serve as independent factors in the prediction prognosis of patients with hepatocellular carcinoma (HCC) (Liang et al. 2020). Although as non-protein-coding RNA molecules, lncRNA plays an irreplaceable role in tumor metastasis with its length longer than 200nt (Li et al. 2016). A majority of study found the function of specific genes involved in ferroptosis (Bersuker et al. 2019; Doll et al. 2019).

With limited comprehensive studies concentrated on ferroptosis-related lncRNAs signatures to predict the overall survival (OS) of patients with head and neck carcinoma, we hold the view that it was necessary and important to construct the ferroptosis-related lncRNAs signature to explore the prediction value of patients with HNSCC by integrated analysis.

Compared with previous researches, we investigated the expression of ferroptosis-related lncRNAs with HNSCC and constructed ferroptosis-related lncRNAs prognostic model to predict the survival staging of patients. In the study, we constructed a ferroptosis-related lncRNAs signature by integrated bioinformatics analyses based on nine ferroptosis-related lncRNAs, which confirmed to have prognostic value and served as independent prognostic factors and potential therapeutic targets of patients with HNSCC.

The nine lncRNAs of the signature are as follows: AC004687.1, AL450992.2, AC010894.2, AL451085.2, AC104083.1, LIPE-AS1, AC108010.1, CTBP1-DT, and PTCSC2. Among them, the nine lncRNAs, AC010894.2 was associated with harmful prognostic effect in HNSCC patients. The other eight lncRNA (AC004687.1, AL450992.2, AL451085.2, AC104083.1, LIPE-AS1, AC108010.1, CTBP1-DT, PTCSC2) had the favorable prognostic effect.

Studies carried out showed that papillary thyroid cancer susceptibility candidate 2 (PTCSC2) was associated with vulnerability to familial origin non-medullary thyroid cancer (FNMTC) (Hińcza et al. 2019) and transcripts of PTCSC2 were downregulated in papillary thyroid carcinoma (PTC) tumors (He et al. 2015). Furthermore, when PTCSC2 binds myosin-9 (MYH9), MYH9 performed to inhibit the promoter activity in a bidirectional promoter shared by FOXE1 and PTCSC (Wang et al. 2017). AC008750.1 was closely associated with oral squamous cell carcinoma (OSCC) in prognostic OSCC patients and showed the value in distinguishing OSCC patients’ survival status effectively (Huang et al. 2020).

Research discovered that AL450992.2 in Th17 cells was the candidate potential biomarker and therapeutic target in patients with autoimmune diseases (Teimuri et al. 2018). Gene expression studies revealed that LIPE-AS1 had an effect on DNA damage, which indicates the potential mechanism in the activity of tumor cell (Goyal et al. 2017).

The remaining five ferroptosis-related lncRNAs (AC010894.2, AL451085.2, AC104083.1, AC108010.1, and CTBP1-DT) showed that there was no relevant report that proved the relationship with cancer out to date. Hence, further researches were needed to investigate the prognosis mechanism of these ferroptosis-related lncRNAs with HNSCC.

There are limitations to this research. Firstly, our research is a retrospective study that needs prospective verification. Secondly, we performed the analysis based only on TCGA-HNSC data, more current real-world data are needed to minimize deviation. Thirdly, the ferroptosis activity has not yet been experimentally validated. Hence, we will improve our findings by further research on experimental analysis to provide more solid evidence on the potential value of ferroptosis-related lncRNAs with HNSCC.

In conclusion, the study identified nine ferroptosis-related lncRNAs and constructed a ferroptosis-related lncRNAs signature based on these nine lncRNAs to predict OS in HNSCC patients, which confirmed to have prognostic value and serve as independent prognostic factors as well as potential therapeutic targets of patients with HNSCC. It provided a new insight for further research of HNSCC.

## Supplementary Information

Below is the link to the electronic supplementary material.

Supplementary file1 (XLSX 10 KB)

## Data Availability

The raw data of this study are derived from the publicly available TCGA database (https://portal.gdc.cancer.gov/).
